# Impact of COVID-19 stress on the psychological health of sexual & gender minority individuals: A systematic review

**DOI:** 10.3389/fgwh.2023.1132768

**Published:** 2023-03-29

**Authors:** Sumona Datta, Tilottama Mukherjee

**Affiliations:** ^1^ Department of Psychology, Government General Degree College, Singur, Hooghly, India; ^2^ Department of Psychology, University of Calcutta, Kolkata, India

**Keywords:** Sexual and gender minority (LGBTQ) populations issues, LGBTQ+, depression, anxiety, COVID-19 pandemic, stress

## Abstract

**Introduction:**

The differential effect of the Covid-19 pandemic on the mental health of the population around the globe is well documented. Social isolation, loss of job, financial crisis, and fear of infection due to the pandemic have widely affected people across countries, and the sexual and gender minority (SGM) group is no exception. However, the additional stressors like stigma, discrimination, rejection, non-acceptance, and violence associated with diverse sexual orientation complicated the situation for the SGM group in the context of the Covid-19 pandemic.

**Method:**

The present study conducted a systematic review of research (*n *= 16) investigating the impact of Covid-19 stress on the psychological health of SGM individuals. The review had two objectives: (a) to explore the effect of the stress associated with the pandemic on the psychological health of the SGM individuals; and (b) to identify potential stressors associated with the Covid-19 pandemic affecting the mental health of SGM individuals. Studies were selected following a PRISMA protocol and several inclusion criteria.

**Results:**

The review provided new insights into the mental health issues of the SGM individual in the Covid-19 context. The outcome of the review focused on five aspects: (a) depression and anxiety symptoms related to Covid-19 symptoms; (b) perceived social support and Covid-19 stress; (c) family support and psychological distress related to Covid-19; (d) Covid-19 stress and disordered eating, and (e) problem drinking and substance abuse associated with Covid-19 stress.

**Discussion:**

The present review indicated a negative association between Covid-19 stress and psychological distress among sexual and gender minority individuals. The findings have important implications for psychologists and social workers working with this population and policymakers around the globe.

## Introduction

The incidence of Covid-19 in the past couple of years caused immense damage to the physical and mental health of people around the globe (1–4). With subsequent waves of the mutant version of the virus, millions of people suffered, and thousands were dead (5–8). The existence of co-morbid physical conditions like cardiac problems, diabetes, cancer, etc. proved to be fatal in most cases. In this mayhem, the marginalized communities suffered significantly under such circumstances, with lessened healthcare delivery and limited social security (9). The lesbian, gay, bisexual, transgender, and queer (LGBTQ) community is one of those marginalized groups that suffered immensely.

To reduce the spread of infection, every government imposed lockdowns, social distancing, quarantine, and banned travel around the world (10, 11). Although the Covid virus affected people around the globe in a similar way, the outcomes were felt differently across some sections of the society, specifically for the SGM population, the reason being a profound level of social discrimination faced by these individuals across different cultures and societies (10). Moreover, studies reported that many of them have co-morbid conditions of HIV, Cancer, and other forms of physical conditions, which increased their difficulties instead (12, 13). Lockdown and quarantine situations also inhibited them from going to their workplace, which in certain cases, serves as an escape for them, especially in low-income countries of Asia and Africa (10, 14). Hence, the overall situation during the pandemic undoubtedly put them at a higher risk of physical and psychological turmoil.

### Risk factors for depression, anxiety, and suicidality among SGM youths

Recent researches suggests that loneliness, anxiety, fear of contraction, and limited access to healthcare facilities due to the Covid-19 pandemic might have worsened psychological health with heightened symptoms of depression, anxiety, self-harm, and suicidal ideation among minority groups (1–4, 15). For sexual minority groups, the situation was more compound because of their pre-existing psychological vulnerabilities associated with social inequalities and discrimination (5–8). Research indicates that the Covid-19 pandemic also resulted in changes in the life circumstances of sexual minority groups. For instance, Conron (16) reported that several SGM young adults in the US had to return to their parental house due to the financial crisis, loss of jobs, and complete lockdown of university campuses due to the pandemic. This imposed additional pressure on them as many of them preferred avoiding their families due to non-acceptance by the family members Across the world, the socially disadvantaged SGM group faced unemployment, food insecurity, social discrimination, and severe financial crisis during the Covid-19 pandemic (17). All this resulted in heightened psychological vulnerabilities and declined subjective well-being among these young adults (5, 8, 18). Pre-existing mental health conditions such as depression, anxiety, and addiction problems, further aggravated the situation for them as they were forced to stay at home with very limited social interactions and rejection from family members (9, 19).

Another line of studies pointed out that alcohol use, substance abuse, and drug dependence among SGM young adults is a common practice (20, 21), even before Covid-19. As noted by Bourdieu (22), subtle differences in “cultural and normative markers” (23) like body language, gestures, accent, etc. can interact with demographic identities like gender, race, and ethnicity to produce hierarchical judgments potentially enough to cause discrimination in the society. Contributing factors to the higher incidence of drug dependence and substance abuse among SGM youths have often been attributed to such hierarchical power structures creating and causing inequalities in employment, health care, living conditions, and other social aspects (24–27). Several pieces of research across the globe reported a higher incidence of substance abuse among SGM youths during Covid-19, leading to poorer mental health and self-harm tendencies. For instance, Slemon et al. (27) compared SGM youths with non-SGM youths in a Canadian population. The study found that in comparison to the non-SGM group, the SGM respondents had experienced a higher impact of Covid-19 and substance abuse, which included poorer psychological health, deteriorated coping, self-harm behaviors, suicidal thoughts, and greater dependence on drugs and substance abuse to survive. Similar findings were reported by Salerno et al. (8) in a US population. The researchers found that almost one-third of the SGM respondents had an increased level of substance abuse since the commencement of Covid-19. This increased abuse along with alcohol problems, and drug dependence contributed to ill-psychological health and complicated the situation among SGM youths.

Higher psychological distress among SGM youths and adolescents has been reported by several researchers. Risk factors for increased suicidality among this minority group result from symptoms of depression, hopelessness, substance abuse, psychiatric distress, and recent incidences of suicide by another family member or a friend (28–30). During the pandemic, an elevated level of loneliness was reported in SGM adolescents and young adults (31, 32). Herrmann et al. (33) compared the loneliness and depressive symptoms among transgender and cisgender individuals across the first and second waves of Covid-19 in a German population. The researchers noted a higher level of loneliness among transgenders in comparison to the cisgender respondents. Moreover, it was found that loneliness mediated the symptoms of depression among the SGM respondents. Jacmin-Park et al. (34) found that the buffering effect of perceived loss of social support due to discrimination during the pandemic on depressive symptoms was four times higher among the transgender group than the cisgender respondents. Gonzales et al. (15) found more frequent symptoms of psychological distress and symptoms of anxiety and depression among 60% of American college-going SGM youths. As per the Sexual Minority Stress Model (35), group solidarity and social support are important protective factors for the SGM group against psychological distress associated with stigma and discrimination (34–37).

Mental health issues among the SGM group associated with the Covid-19 pandemic are a crucial concern, although under-explored. Several studies have been conducted to explore the issue in the past couple of years. The present study intends to present a systematic review of the incidences of psychological distress as reported among SGM youths, especially symptoms of depression and anxiety, associated with the isolation and discrimination faced during the Covid-19 pandemic across the world. The review has the following objectives: (a) To explore the impact of Covid-19 stress on the psychological health of the sexual minority group, and (b) To identify the stressors associated with the Covid-19 pandemic that impacts the mental health well-being of the sexual minority individuals. This review will help integrate and understand the overall effect of the Covid-19 pandemic on the psychological health of the SGM youths.

## Method

### Search method

**Figure 1:**
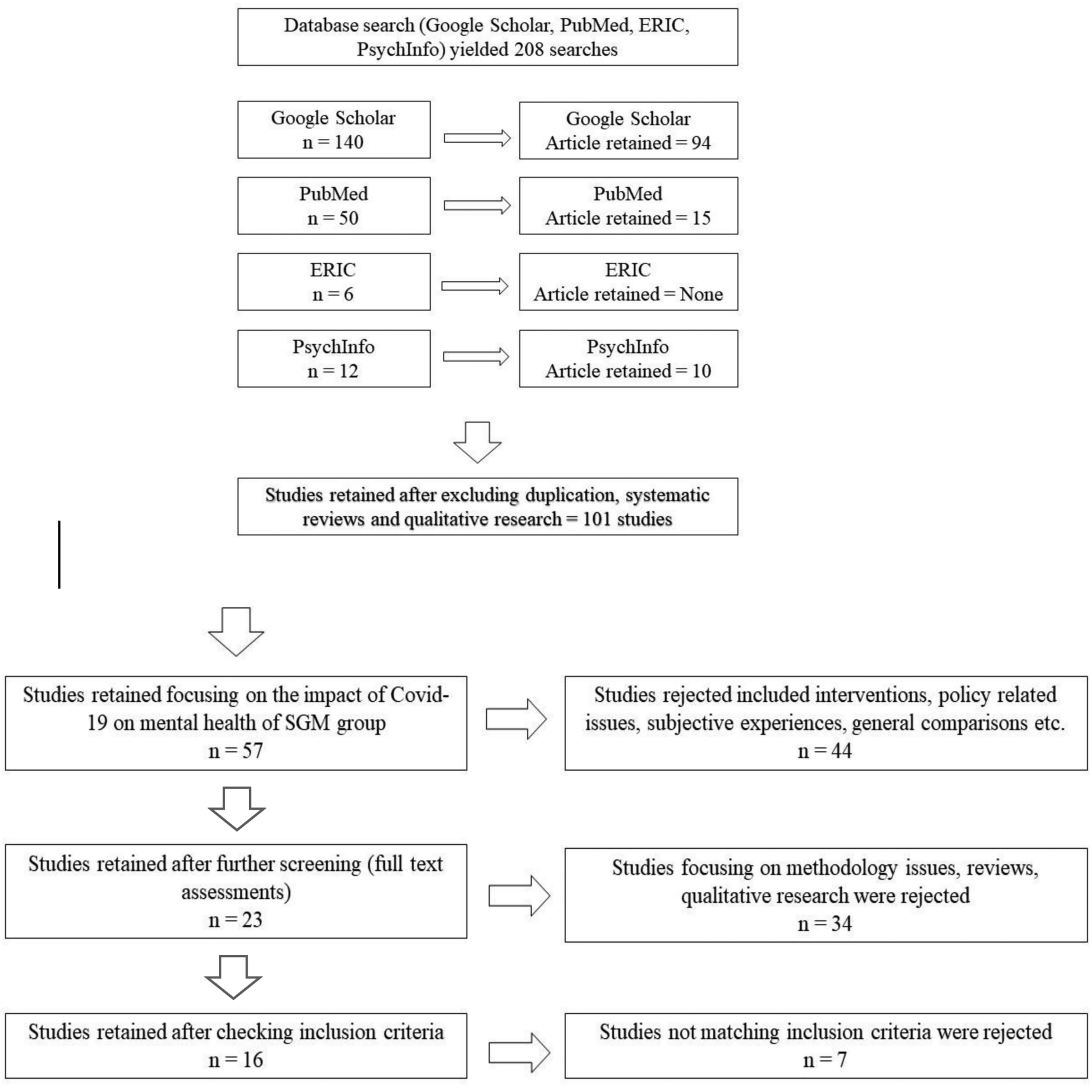
PRISMA framework representing the selection process.

The review followed the format of the PRISMA (Preferred Reporting Items for Systematic Reviews) framework. The selection process is illustrated in Figure 1 which depicts the selection process following the inclusion criteria for the review. Chiefly, studies were selected from online databases namely, Google Scholar, PubMed, Eric, and PsychInfo. The keywords used for the search were “Sexual Minority”, “LGBTQ” “Transgender”, “Mental Health”, “Covid-19”, “Pandemic”, and also “Depression” and “Anxiety”, jointly and also in isolation. The review was done during the second week of September 2022. The initial search generated thousands of research out of which 140 searches were relevant in Google Scholar, 50 searches were relevant in PubMed, ERIC yielded six related searches, and PsychInfo yielded 12 searches. Relevance was judged based on the keywords used for the search. For instance, a combination of “Mental Health”, “Covid-19”, “Pandemic”, “Depression” and “Anxiety” keywords yielded hundreds of studies addressing populations other than sexual minority groups. Again, a search with the keyword “Sexual Minority”, and “LGBTQ” yielded research addressing different issues related to this population. Hence only those studies addressing the impact of the “Covid-19” “pandemic” on the “Mental Health” of the “Sexual Minority”/“LGBTQ”/“Transgender” population were included in the current review, yielding a total of 208 studies.

Figure 1 shows the PRISMA framework used for the search process. out of the relevant 208 searches yielded by the database search, 101 studies were retrieved (excluding the duplication).

In the next stage, the 101 studies were reviewed and 57 studies were selected focusing on the impact of Covid-19 on the mental health of the SGM group. The 44 studies rejected in this stage were focusing on intervention, policy-related issues, subjective descriptions of experiences, systematic reviews, general comparisons, and others. This review process was mostly done by analyzing the study abstracts and occasionally looking into the full paper as required. In the next stage, out of the 57 studies, 23 studies were selected after rejecting other studies focusing on methodology issues, comparisons, qualitative research, etc. Studies focusing on subjective descriptions, methodological issues, and systematic reviews were excluded since most of those addressed issues beyond the purview of the current study. Qualitative researches were also excluded because comparing results across quantitative reports using questionnaires with qualitative reports based on interviews is complicated and might invite unnecessary methodological complications.

Subsequently, the 23 studies (full papers) were thoroughly reviewed and finally, 16 studies were selected following the inclusion criteria: (a) Study subjects belonging to Sexual and Gender Minority groups; (b) Subjects are adults; (c) Subjects experiencing symptoms of depression and anxiety; (d) Study reported quantitative assessment (using questionnaires instead of semi-structured interviews); (e) Sample characteristics clearly mentioned; (f) Study objectives focusing on the impact of Covid-19 on the psychological health of SGM group; (g) Details of testing materials discussed clearly;(h) Statistical analysis reported and discussed elaborately; (i) Results indicate a significant impact of the pandemic on the psychological health of SGM group. These 16 studies were independently reviewed by the two authors on these inclusion criteria and then included in the review. All the studies fulfilled the inclusion criteria set for the review and are therefore considered for the present review.

### Quality assessment

All the 16 studies were quality assessed using the Joanna Briggs Institute (JBI) checklist for analytical cross-sectional studies (38). The checklist consists of eight criteria assessing the quality of the studies included (related to methodological issues). The studies were rated on the checklist by both authors independently. All the studies were found to be meeting the standards of quality assessment.

## Results

### Study characteristics

The studies selected for the review, mostly followed online or web-based cross-sectional survey method, comparing the different sexual minority groups with cisgender heterosexual groups, except three studies that followed a Cohort design/ Longitudinal Cohort study (39–41). For instance, Kamal et al. (41) considered the data collected during Wave 1 of the Covid-19 pandemic through a US-based longitudinal cohort study (CARES: Covid-19 Adult Resilience Experiences Study) for the investigation. Similarly, Chang et al. (40) collected data in three cohorts in 2020, January–March (*n *= 99), July–September (*n *= 390), and September–November (*n *= 305). The second and third cohorts were finally considered for the investigation. On the other hand, the study by Bécares and Kneale (39) was a typical longitudinal cohort research that was a part of the Millennium Cohort Study done among UK infants born between 2000 and 2002. The present research included participants who had responded in the 6th and 7th data collection sweeps and during the 1st wave of the Covid-19 pandemic. Hence, this study provided a comparative view of the mental health of the SGM group during the pre-pandemic phase and the 1st wave of the pandemic. Apart from these three studies, one other study (42) followed a mixed method design, where a quantitative assessment was mixed with a qualitative approach and hence aided in providing a more comprehensive and rich understanding of SGM mental health issues during Covid-19 pandemic. Additionally, the study by Gato et al. (6, 7) did a cross-country exploration among LGBTQ+ individuals from six different nations. This study, therefore, provides a more in-depth insight into the prevalence of psychological distress among the SGM group across different nations. It is important to note that all the studies followed a web-based survey method, considering the physical restrictions imposed during the pandemic. Table 1 provides a comparative view of the study characteristics and demographics of the study participants of all sixteen studies included in the review.

**Table 1:** Comparative view of the study characteristics and demographics of the study participants.

Author(s) with year	Study design	Sample size	Age	Sexual orientation	Racial/ethnic group
Oren (43)	Correlational Study	157 Adults	Mean age = 26 years	Lesbian, Gay, Bisexual	Israeli population
Akre et al. (44)	comparative study using quota sampling method	3,245 adults	18 years or older	Transgender and cisgender	US population
Kneale and Bécares (45)	Cross-sectional Web based survey	310 adults	18 years or older	Lesbian, Gay, Bisexual, Queer, having another minority sexual minority, transgender people.	UK population
Moore et al. (46)	Online Cross-sectional Study	1,380 respondents (290 SGM adults and 1,090 cisgender heterosexual adults)	Mean age of SGM respondent = 34.26 years; Mean age of cisgender group = 46.12 years	15.2% Lesbian, 18.3% Gay, 50.7% Bisexual, 22.1% Queer, 5.5% Heterosexual, 12.4% Asexual,.7% Calibate, 6.2% decline to answer	US population (>90% white population, rest included Black, Asian, Alaskan native, Hispanic, Hawaiian native and others)
Suen et al. (47)	Community based online survey	857 adults	55% aged between 16 and 25 years, 33.6% between 26 and 35 years, 11.4% aged above 36 years.	61.1% Lesbian/Gay, 32.2% Bisexual or Pansexual, and 6.7% others (queer, asexual, etc.)	Hong Kong population
Gato et al. (7)	Online survey	403 respondents	Age range = 16–30 Mean age: 22.13 years	56.6% Lesbian/Gay, 27.9% Bisexual, 11.7% Pansexual, 1.2% Asexual, 1.7% Heterosexual, 1% Other (Queer)	Portuguese population
Bécares and Kneale (39)	Cohort Study with longitudinal data	2,211 respondents	Mostly aged 19 years during data collection	Heterosexual and Sexual Minority respondents (detailed category not considered due to small cohorts)	UK population (86% white, 4.5% mixed and 9.5% Indian, black, Pakistani or Bangladeshi and others)
Urzúa et al. (48)	Cross-sectional study under an observational design	1,181 respondents	18–64 years; Mean Age_Homosexual _= 31.5 years; Mean Age_Bisexual _= 24.5 years; Mean Age_Other Sexual orientation_ = 25 years	64% identified as homosexual, 23% identified as bisexual, 13% identified as having other sexual orientation (pansexual, demisexual, asexual, etc.)	Chilean population
Chang et al. (31)	Cohort Study	695 sexual minority adults	Age range = 18–29 years; Mean age = 23.09 years	50.2% Lesbian/Gay; 49.8% Bisexual, Pansexual and others	US population; 60.6% white, 12.5% Latin, 10.5% Asian, 8.3% Black, 7.3% Biracial or multiracial, 1.2% others.
Kamal et al. (32)	Longitudinal Cohort Study	981 adults	Mean age = 24.37 years	63.6% Heterosexual and 32.6% Sexual Minority group	US population; 60.8% white, 20.9% Asian, 6.3% Mixed, 5.8% Hispanic, 4.8% Black, 1.5% others.
Salerno et al. (8)	Cross-sectional study	294 sexual minority young adults and 894 non-sexual minority young adults	Age Range = 18–26 Mean age = 22 years	Heterosexual, Bisexual (Lesbian/Gay), Other	Sexual minority group: 55.4% white,.07% Latino,.06% black, 11.56% Asian, 17.7% Multiracial,.03% Other
Hart et al. (49)	Online Survey	830 LGBTQ + adults	Age range = 18–30 years; Mean age = 20.6 years	36.4% Bisexual, 24.5% Gay, 14.8% multiple orientation, 7.2% Queer, 6.3% Asexual, 0.7% Heterosexual, 1.3% Other, 8.3% Unsure	US population (69.9% White, 10.1% Multiracial, 8.3% Asian, 7.5% Latin, 2.2% Black, 1.2% Other, 0.7% preferred not to say)
Goodyear et al. (50)	Cross-sectional survey	502 LGBTQ2 + adults	Age Range = 18 years and above; 28.3% aged 18–34 years, 41.8% aged 35–54 years, 29.9% aged above 55 years	Lesbian, Gay, Bisexual, Transgender, Queer, Two-Spirit etc.	Canadian population; 69.2% of European origin, 25.5% having non-European origin, 5.4% of indigenous origin
Tabler et al. (33)	Convergent mixed method design	411 LGBTQ + adults	Age range = 18–86 years; Mean age = 28.5 years.	71% Heterosexual (56% women), 29% LGBTQ + individuals	US population (87% White, 13% Latinx).
Gato et al. (6, 7)	Cross country exploratory research	1,934 LGBTQ + young adults	Age range = 18–29 years; Mean age = 22.70 years	52% Lesbian/Gay, 32.3% Bisexual, 5.0% Pansexual, 1.8% Asexual, 1.3% Heterosexual, 7.5% Other (Queer etc.)	Participants from six countries; Portugal: 18.6%, UK: 5% Italy: 5.5% Brazil: 32.2% Chile: 37.0% Sweden: 1.8%
Fish et al. (5)	Cross-sectional Online survey	2,996 adults (18.06% sexual minority)	Mean age = 32.20 years	81.94% Heterosexual, 0.04% Lesbian/Gay, 11.58% Bisexual, 0.03% Other.	US population; 64.49% White, 6.48% Latina/o/x, 7.08% Black, 11.95% Asian American, 9.71% Multiracial, Multiethnic or other

### Sample characteristics

Out of the 16 studies, seven studies presented a comparative account across heterosexual and SGM respondents (5, 8, 39, 32, 33, 44, 46), while three more studies included heterosexual respondents although in very small percentages (6, 7, 49). The rest of the studies included only the SGM group for the investigation. Participants of all the studies were adults with mostly young age groups included in the research. Additionally, the studies considered for the review include participants from across a wide range of race/ethnic groups. Eight studies were conducted on the US population (5, 8, 31–33, 44, 46, 49), although the US population included in the studies was a heterogenous mix of Asian American, Latin American, African American, and multiracial origins along with White Americans. Two studies were conducted among the UK population (39, 45), also a heterogeneous group of white individuals, mixed racial groups, and Asian individuals. The rest of the studies included participants representing several different countries like Israel (43), Hong Kong (47), Portugal (7), Chile (48), and Canada (50). Gato et al. (6, 7) included participants from six different countries namely, Portugal, the UK, Italy, Brazil, Chile, and Sweden. Hence, the study results are representative of the mental health scenario of the SGM group across a wide geographical region.

### Study measures

All the studies included in the review assessed the psychological distress related to Covid-19 and the associated symptoms of depression and anxiety among SGM individuals. While the measures for assessing depression and anxiety were all standardized instruments (for instance, 8-item Patient Health Questionnaire (PHQ-8), Kroenke, et al. (51) and 7-item Generalized Anxiety Disorder scale (GAD-7) (52); Depression Anxiety Stress Scales (DASS-21), Lovibond and Lovibond (53), most studies used customized questions to assess the Covid-19 related stress (for instance (31, 43, 46, 47, 50). The chief reason cited for this practice was a lack of a standardized scale for measuring stress related to Covid-19 among SGM individuals, although Tabler et al. (33) used the Pandemic Stress Scale developed by Taylor et al. (54).

Apart from anxiety, depression, and psychological distress, several studies also measured perceived social support (39, 32–43, 46). The most commonly used measure was the modified Medical Outcomes Study Social Support Survey (8-items) (55), although Kamal et al. (32) and Oren (43) used the 12-item Multidimensional Scale of Perceived Social Support (57) for measuring the same. Some of the studies also studied variables like Loneliness, Rumination, and Quality of relationships in addition to the stress and psychological symptoms related to Covid-19. Additionally, Hart et al. (49) and Tabler et al. (33) measured eating disorder symptoms as well using the Eating Disorder Examination-Questionnaire (58) and Eating Disorder Examination—Questionnaire Short (EDE-QS) (12 items) (59, 60) respectively. Akre et al. (44) and Goodyear et al. (50) measured problem drinking using the Patient-Reported Outcomes Measurement Information System (PROMIS) and substance use through a set of semi-structured questions. Table 2 presents a detailed account of the measures used in the studies.

**Table 2:** Comparative view of the study measure and study outcomes.

Author(s) with Year	Study Variables	Study Measures	Study Outcomes
Oren (43)	COVID-19 stress, internalized homophobia, concealment, need of acceptance, social support, anxiety, and depression.	Stress related to COVID-19 was assessed by asking four questions developed by the author; Internalized homophobia was measured by the 9-item Internalized Homophobia Scale; Concealment motivation and Acceptance Need were measured by the three-item Concealment Motivation subscale and the five item Acceptance Need subscale of the Lesbian, Gay and Bisexual Identity Scale (61), 12-item Multidimensional Scale of Perceived Social Support (MSPSS; Zimet et al. 1988) for measuring Social Support; Four-item Symptom Checklist Anxiety and six-item Symptom Checklist Depression scales (62) were used for measuring Anxiety and Depression.	The findings stressed upon the significance of minority stress, focused on the psychological mediation framework, and emphasized the need to study the differential impact of stress on the psychological health of SGM individuals.
Akre et al. (44)	Depression, anxiety, and problem drinking	Patient-Reported Outcomes Measurement Information System (PROMIS) measures to assess depression, anxiety, and problem drinking during the COVID-19 pandemic (63).	LGBTQ1 communities reported poorer psychological health and more frequent problem drinking than the cisgender heterosexual respondents during the COVID-19 pandemic.
Kneale and Bécares (45)	Perceived stress, Depression	4-item Perceived Stress Scale (64); 10- item Center for Epidemiological Studies Depression scale (CES-D-10) (65).	Findings revealed that poorer mental health among SGM individuals could be partially accounted for by experiences of social discrimination, the latter having a debilitating impact on the subjective well being of the SGM individuals.
Moore et al. (46)	Physical symptoms, psychological symptoms, rumination, and perceived social support.	COVID-19-related self-report items, Sexual and gender identity Questions; 21-item measure asking about a variety of COVID-19 and non-COVID-19 related physical symptoms; 8-item Patient Health Questionnaire (PHQ-8) (51); 7-item Generalized Anxiety Disorder scale (GAD-7) measure of anxiety symptom (52); 19-item Medical Outcomes Study Social Support Survey (MOS4) (66); 10-item Ruminative Response Scale (66).	It was noted that SGM respondents had more often experienced COVID-19-related bodily symptoms and psychological symptoms of depression and anxiety.
Suen et al. (47)	COVID 19 stressors, Depression, Anxiety	Questions on COVID-19-related stressors, 9-item Patient Health Questionnaire (PHQ-9) for depression (Kroenke et al. 2001), 7-item Generalized Anxiety Disorder for anxiety (52).	Findings revealed that the Covid-19 related stressors experienced by the SGM individuals, in addition to the general stressors associated with the pandemic, mediated the symptoms of depression and anxiety among them.
Gato et al. (2020)	Depression, anxiety, and Stress	Portuguese version of the Depression, Anxiety and Stress Scales 21-Item Version (DASS-21) (67, 68).	Findings revealed that the association between the pandemic's individual impact and both depression and anxiety are partially mediated by family climate.
Bécares and Kneale (39)	Social support, Self-rated health, psychological distress, anxiety, loneliness	Questions related to Sexual Orientation, Kessler 6 scale for psychological distress (Kessler, 2006); Generalized Anxiety Disorder (2 items) questionnaire (Kroenke et al. 2007); UCLA Loneliness Scale, Short Social Provisions scale (3 items) (69).	Findings reported significantly poor social support among SGM young adults, worse self-rated health, and more severe psychological distress, anxiety, and loneliness in comparison to heterosexual counterparts.
Urzúa et al. (48)	Anxiety, Depression, Stress	Spanish version of Depression Anxiety Stress Scales (DASS-21) was used to measure Anxiety, Depression and Stress (70); Lovibond and Lovibond, 1995a; 1995b; (71).	Poorer mental health was observed among the bisexual and other sexual orientations (pansexual, demisexual, asexual) group as opposed to gays and lesbians. Findings suggest a higher occurrence of depressive symptoms and anxiety, related to stress. Moreover, bisexual women presented a higher prevalence of symptoms related to psychological distress than men. A similar trend in anxiety symptoms was observed among lesbians, as compared to gay individuals.
Chang et al. (31)	Depression, Negative Impact of COVID-19	8-item version of the Patient Health Questionnaire (PHQ-8) (51), Negative Impact of COVID-19 assessed by one question with ratings from 1 to 10.	Results indicated that transgender or gender-diverse women who are full-time students are at a higher risk of getting affected by the pandemic. Moreover, the study confirmed the association between the negative effect of Covid-19 and depressive symptoms at a two-month follow-up.
Kamal et al. (32)	Perceived social support; Lifetime discrimination; severity of COVID-19-related worries and COVID-19- related grief; Depression; Anxiety, PTSD symptoms.	12-item Multidimensional Scale of Perceived Social Support (MSPSS) for measuring Perceived social support (55).; Lifetime discrimination was assessed using the 11-item Lifetime Discrimination Scale (Williams et al. 1997); Two 6-item scales, that have been used in previously published work, assessed the severity of COVID-19-related worries and COVID-19- related grief (Liu et al. 2020a; 2020b); 8-item version of the Patient Health Questionnaire (PHQ-8) for Depression (Kroenke et al. 2001); 7-item version of the Generalized Anxiety Disorder Scale (GAD-7) for Anxiety (52); 17-item version of the PTSD Checklist—Civilian Version (PCL-C) for PTSD symptoms.	Study findings reported significantly more severe symptoms of depression and PTSD as also COVID-19-related worries and grief among the SGM respondents in comparison to the non-SGM counterparts. The effect persisted even after controlling for the effects of family support, lifetime discrimination, and pre-existing mental health diagnoses.
Salerno et al. (8)	Psychological distress, well-being, pre-and post-onset of COVID-19 living circumstances.	6-item Kessler-6 (K6) (72) psychological distress scale measured nervousness, hopelessness, restlessness, depression, worthlessness, and whether everything is an effort within the past 30 days; Cantril Ladder measured self-rated well-being (73); pre-and post-onset of COVID-19 living circumstances were measured with specific questions.	Findings revealed that SMYAs who went back to their parents’ residence after the onset of COVID-19, reported higher levels of psychological distress and lower levels of psychological well-being, in comparison to those who were already staying with their parents both pre- and post-onset of COVID-19 pandemic.
Hart et al. (49)	Eating behavior, Quality of relationship, Psychological stress symptoms	A brief and modified version of the COVID-19 Adult Experiences and Psychological Symptoms Questionnaire (74); Quality of Relationships Inventory (QRI)-support scale (75); Dietary Restriction Screener 2 (DRS-2) (76); Eating Disorder Examination-Questionnaire (57).	The study reported minor but significant association among variations in average disordered eating behaviour severity and interpersonal relationships, average quality of relationships in the home, and staying with someone not accepting one's identity.
Goodyear et al. (50)	Substance abuse, Coping, Self-reported change in mental health, Suicidal thoughts	Substance use was assessed by asking participants to “indicate how your use of any of the following has been impacted by the COVID-19 pandemic”, including “Drinking alcohol” and “Use of cannabis products”. Response options included “More”, “Less”, “No change”, “Not applicable”, and “Prefer not to say”. Respondents who indicated “More” were classified as having increased their use of the respective substance, whereas those who indicated “Less”, “No change”, and “Not applicable” were classified as not having increased their substance use. Coping was assessed through the question, “Overall, how well do you think you are coping with stress related to the COVID-19 pandemic?” Response options “Not very well” and “Not well at all” were classified as poor coping, whereas responses “Very well” and “Fairly well” were classified as not poor coping. Self-reported change in mental health was also assessed by asking participants, “Compared to before the COVID-19 pandemic and related restrictions in Canada, how would you say your mental health is now?” Response options “Slightly worse now” and “Significantly worse now” were classified as experiencing worse mental health, whereas responses “Significantly better now”, “Slightly better now”, and “About the same” were classified as not experiencing a deterioration in mental health. Suicidal thoughts were then assessed through a question asking whether participants had “Experienced suicidal thoughts/feelings” within the past two weeks.	Researchers noted that increased alcohol use among 24.5% of the respondents and 18.5% reported increased cannabis use due to the pandemic. Furthermore, they found that higher levels of alcohol use were associated with worse coping skills and poor self-reported psychological health. On the other hand, increased use of cannabis was found to be related to suicidal thoughts.
Tabler et al. (33)	Eating disorder, Pandemic stress, LGBTQ Identity, Resilience, Social Support	Eating Disorder Examination—Questionnaire Short (EDE-QS)(12 items) (48, 49); Pandemic stress scale (5 items) (44), LGBTQ + identity combines information from self-reported sex assigned at birth, gender identity, and sexual identity, to create a 3-category measure comparing cisgender and heterosexual (cishet) women, and LGBTQ + identifying participants, to cishet men, Brief Resilient Coping Scale (4-items) (77), modified Medical Outcomes Study Social Support Survey (8-items) (45).	Study results suggest that LGBTQ + individuals are more vulnerable to experiencing a uniquely increased level of pandemic-related stress. Also, the stress related to the pandemic is associated with increased symptoms of eating disorders and elevated risk of perceived weight gain, with almost 1 in 3 participants reporting clinically significant symptoms of eating disorders. Moreover, social support, as opposed to resilient coping, was found to be a potential protective shield against elevated symptoms of the eating disorder.
Gato et al. (6, 7)	Psychosocial effects of the COVID-19 pandemic, Depression, Anxiety and Stress	Psychosocial effects of the COVID-19 pandemic (7 items) (Gato et al., 2020), Depression, Anxiety and Stress Scales 21-Item Version (DASS-21) (66); and sociodemographic questionnaire were used.	Younger, non-working participants, living in Europe, and those reporting feeling uncomfortable, isolated, and more emotionally distraught by the pandemic, experienced higher levels of depression and anxiety. Furthermore, depression was predicted by not having higher education, while anxiety was predicted by being isolated at home, having to stay with parents, and worries about contraction.
Fish et al. (5)	Mental health, physical health, quality of life, Psychological distress, Loneliness, Alcohol use, Sexual Identity.	Self-reported mental health, physical health, and quality of life were each assessed using a single item adapted from the Centers for Disease Control and Prevention's “Healthy Days Measure” (78); Psychological distress was assessed using the Kessler 6 (79); Loneliness was assessed using four items from the UCLA Loneliness Scale (80); Alcohol use was assessed with two items; Sexual identity was assessed by asking objective questions.	Findings revealed consistent patterns of deterioration in psychological well-being across the SGM subgroups, nonetheless, variations in mental health, physical health, quality of life, stress, and psychological distress were more profound among the SGM adults in comparison to the heterosexual adults.

### Study outcomes

Almost all the studies highlighted the significance of the impact of Covid-19 related stress on the psychological health of minority individuals. It can be noted that the studies identified potential Covid-19 stressors that contribute to an increased level of psychological distress among SGM individuals.

### Depression and anxiety symptoms related to COVID-19 stress

Increased levels of anxiety and depression were found to be associated with the perceived stress related to the Covid-19 pandemic. Most of the researchers reported such elevated symptoms during the first wave of the pandemic and also during the subsequent waves. Severe symptoms of depression and anxiety were also found to be associated with Covid-19 related physical symptoms (46), psychological distress (c), loneliness (5, 39), and poor quality of life (5). Kamal et al. (32) additionally, reported symptoms of post-traumatic stress disorder associated with higher levels of depression among SGM youths.

Most commonly such experience of psychological distress was attributed to stressors like perceived social support (39, 32–43, 46), family-related issues (8), social discriminations (32) and non-affirmation of one's identity (43).

### Perceived social support and COVID-19 stress

In connection to the issue of social support, Meyer (35) proposed the Minority Stress Model in association with lesbian, gay, and bisexual (LGB) health. As per the minority stress model, stressors can be distal or proximal. Distal stressors are characterized by the actual experiences of discrimination, prejudices, harassment, and even violence, while the proximal stressors include the occurrence of internalized homophobia, the need for acceptance by others, and the suppression of one's own sexual identity (43). Several researchers have provided evidence for the fact that such minority distal and proximal stressors lead to elevated levels of psychological distress, a relationship mediated by the development of pathological cognitive processes, and conflictual social and interpersonal relationships (43, 81). In contrast, perceived social support works as a buffer against the negative effects of minority stressors and therefore can be considered an important resource in protecting SGM mental health (35, 43, 82).

Tabler et al. (33) noted that social support plays an important role as a potential protective safeguard against the elevated risks of disordered eating symptoms associated with Covid-19 stress. These findings were further validated by the work of Bécares and Kneale (39) who noted a significantly poorer level of perceived social support associated with lower levels of self-rated health and poor psychological health among the SGM respondents. Moore et al. (46), in a comparison study among SGM and non-SGM adults, noted significantly lower perception (*p < .001*) of emotional support, tangible support, affectionate support, and positive social interaction support among the SGM respondents in comparison to their non-SGM counterparts. The study also reported significantly heightened symptoms of depression and anxiety among the SGM adults in comparison to the non-SGM respondents. Such findings, therefore, suggest that lack of perceived social support acted as a potential stressor for the SGM individuals under the impact of the Covid-19 pandemic.

### Family support and psychological distress related to COVID-19

There is no dearth of studies reporting a lack of family support among sexual minority groups (32, 83, 84). Kamal et al. (32) found that SGM respondents experienced a significantly decreased level of family support (*p* < .001) in comparison to the non-SGM respondents, and this in turn is associated with significantly increased levels of depression (*p* = .003), post-traumatic stress disorder symptoms (*p* = .013), higher levels of worries related to Covid-19 pandemic (*p* < .001) and grief related to the pandemic (*p* = .032) among the sexual minority individuals than among the non-SGM individuals. Moreover, the mandate of staying at home due to quarantine had a more debilitating effect on the mental health of the minority group (85, 86). Gato et al. (7) noted that individuals staying at home might be experiencing elevated risks of depression and anxiety, provided the fact that the associated home climate is hostile and non-affirming of the sexual identity of the individuals. Hence, staying at home although, provided a sense of security to most individuals during the pandemic, for the SGM group the response was different, owing to the associated discrimination and parental rejections. These findings were also confirmed by Salerno et al. (8). The researchers noted that sexual minority youths who went back to their parental house during the post-onset of the pandemic experienced significantly higher levels of psychological distress and decreased well-being in comparison to the others who were already staying before, the reasons being the same.

### COVID-19 stress and disordered eating

Covid-19 stress is often associated with weight stigmatizing in social media messaging (87), which in turn can contribute to increased perceptions of weight gain and disordered eating behavior (88, 89). Disordered eating behavior among sexual minority groups is a common problem and is often associated with negative experiences of stigma and discrimination (90–92). Such problem behavior is considered a coping mechanism for minority individuals in the face of minority stress (33).

The current review also found that Covid-19 stress among SGM individuals is associated with elevated risks of disordered eating behavior and increased perception of weight gain among them (33). Hart et al. (49) noted that most of their study participants reported an increase in the urge and frequency of engaging in disordered eating behavior. Moreover, they found an association between disordered eating behavior and increased levels of psychological stress due to Covid-19 and the quality of relationships at home and non-acceptance of one's sexual identity by family members. Tabler et al. (33) noted that nearly one out of three participants presented clinically significant symptoms of disordered eating.

### Problem drinking and substance abuse associated with COVID-19 stress

Sexual and gender minority individuals are at an increased level of abusing substances and alcohol in comparison to non-SGM individuals even before the onset of the pandemic (16, 93–95). More often, negative experiences of psycho-social stresses contribute to heavy alcohol consumption and other substance abuse among SGM individuals, which in turn leads to elevated risks of psychological distress (96, 97).

In the present review, Goodyear et al. (50) reported increased alcohol use among 24.5% of the study participants and increased cannabis use among 18.5% of the respondents due to the pandemic stress. furthermore, they reported that higher levels of alcohol consumption were associated with poor coping skills, and lower levels of self-rated psychological health, while increased use of cannabis was associated with suicidal thoughts. Along the same line, Fish et al. (5) reported that in comparison to heterosexual men, gay men, and bisexual men experienced a significant decrease in psychological health, quality of life, higher levels of stress, feelings of loneliness, and alcohol consumption. Lesbian individuals also reported significantly higher levels of alcohol consumption in their study.

## Discussion

The present review had two objectives; first, to explore the impact of Covid-19 stress on the psychological health of the sexual minority group, and, second, to identify the stressors associated with the Covid-19 pandemic. For the same, 16 studies were selected following the PRISMA framework from among an initial pool of 208 studies. The studies were all selected strategically following nine different inclusion criteria. A detailed review of the studies helped fulfill the two objectives of the current study.

All sixteen studies reported an elevated risk of depression and anxiety symptoms among sexual minority individuals. The heightened symptoms of depression and anxiety were also found to be associated with loneliness (5, 39), symptoms of post-traumatic stress disorder, worries related to the Covid-19 severity and related grief (32), poor quality of relationships (49), suicidal thoughts (50), substance use and alcohol abuse (5, 50), and disordered eating behavior (33, 49). Higher levels of depression and anxiety are more often experienced by non-working, European SGM youths, who are more susceptible to feeling uncomfortable, isolated, and emotionally impacted by the pandemic (6, 7) and among transgender or gender-diverse women who are full-time students (31). Urzúa et al. (59) studied the differential impact of Covid-19 stress among the different subgroups of SGM individuals as well. Their findings suggested a higher occurrence of depressive symptoms among bisexual and lesbian women in comparison to bisexual and gay men.

In most cases, the incidences of worsened psychological health and problem behavior were found to be associated with lower levels of perceived social support, discrimination, hostile family climate, and non-affirming of one's sexual identity (5–7, 32–43, 46). Such findings are in line with other similar studies reporting the role of psychosocial factors in mediating the worsened psychological health among SGM individuals (9, 16, 17).

A second objective of the review was to identify the stressors associated with the pandemic. The present review identified two potential stressors, strong enough to produce a debilitating impact on the mental health of the sexual and gender minority population. First, the present review noted the importance of perceived social support as a buffer against the ill effects of the minority stressors like stigma, discrimination, violence, non-acceptance, and rejection (39, 33, 46). Lower levels of perceived social support (emotional, tangible, affectionate, and positive social interactions) are associated with poor psychological health of SGM individuals. This finding is particularly important since this validates the hypothesis of “Social Support as a buffer against stress” in the Covid-19 context. Second, the review identified the importance of family support in mediating psychological well-being among SGM individuals. The findings revealed that decreased level of family support is associated with increased symptoms of depression, anxiety, post-traumatic stress disorder, and higher levels of worries and grief related to the Covid-19 pandemic (32). Moreover, similar effects on mental health resulted from a hostile family environment and non-acceptance of one's own sexual identity by the family (7, 8). Hence, a lack of family support can be considered a potential stressor in the Covid-19 context.

## Implications of the study

Although there has not been any dearth of studies reporting the negative impact of the Covid-19 pandemic on the mental health of the sexual and gender minority group, a review of the articles was lacking in this post-Covid period. A review helps gain new insight into an ongoing event or situation. After the onset of Covid-19, there have been several reviews done, focusing on different aspects of the impact of Covid-19 stress (98–101). However, only a handful of reviews focused on the mental health of the sexual and gender minority group (101–103).

Overall, the present review highlighted the importance of studying and considering the mental health outcomes of Covid-19 stress for the sexual and gender minority group. Some of the previously done reviews also underlined the same, although in a different context, for instance, (101) studied the application of psychotherapy for the minority population in the Covid-19 context. However, the present review not only summarized the negative impact of the pandemic stress on the psychological health of the SGM group but also identified the potential stressors and associated outcomes for the minority individuals in the Covid-19 context. This is one of the major strengths of the present review and contributes to the relevance of the study in the present context. Greater effort must be put on the part of clinical psychologists and social workers to mitigate the debilitating impact of the pandemic on the mental health of sexual and gender minority individuals. Moreover, policymakers must consider the unique impact of psychosocial factors on SGM individuals while developing any mental health policy or stating a law applicable to this population.

Another strength of the present review lies in the fact that the review included studies of several different populations, mostly representing the European, British, and US populations. One of the studies also included the population from Hong Kong (47), providing a representation from one of the Asian countries. This is particularly important because this points to the fact that the impact of Covid-19 stress on the psychological health of the SGM group remains indifferent across different cultures, societies, and even continents.

The timing of the review is also of particular importance and has implications. The present review has been done at such a time when most countries have overcome the three waves of the pandemic and the impact of the pandemic on the mental health of people around the globe is more or less explored. All the reviews done previously (during the ongoing first wave or second wave) could only provide a partial, and in certain cases, an incomplete picture of the scenario. The present review is well-timed and provided a well-representation of the situation across different continents, countries, and cultures. Hence, the findings have wider implications for clinical psychologists, social workers, and policymakers around the globe.

## Limitations and future research

Like any other research, the present review also has some limitations. First, the present study included SGM individuals mostly belonging to the young adult age group. However, the pandemic has also hugely affected the SGM older adults and individuals with some terminal conditions like HIV infected population. But researches reporting this impact were beyond the purview of the present review, the reason being that such studies are quite less in number and hence do not qualify for a full-fledged review. Future research should consider an elaborate review of such issues in the context of the Covid-19 pandemic. Second, almost all the studies included studied the European, Canadian, American, and British populations. Only one Asian study done on the Hong Kong population could be included. It would have been better if more studies from the Asian continent and African continents could be included in the present review. A major reason for this has been a lack of empirical studies done on the SGM population of Asian or African countries during the pandemic. This can be attributed to the limitations in the healthcare facilities and research infrastructure of these developing societies, in comparison to western societies. Future research should take note of this issue and consider having more research focusing on SGM individuals in developing societies.

## Conclusion

The present study reported a systematic review of sixteen studies focusing on the impact of Covid-19 stress on the mental health of the sexual and gender minority population. The review of the studies reported the association between the negative effect of the pandemic stress and higher levels of psychological distress, symptoms of depression, anxiety, loneliness, problem drinking, and disordered eating behavior among minority individuals. Moreover, the study also identified the lack of perceived social support and family support in mediating the inverse relationship between increased Covid-19 stress and decreased psychological health of the sexual and gender minority individuals. Overall, the review provided a detailed understanding and newer insights into the mental health issues of the sexual and gender minority group in the context of the Covid-19 pandemic.

## Data Availability

The original contributions presented in the study are included in the article/Supplementary Material, further inquiries can be directed to the corresponding author/s.
